# Complete Genome Sequence of Bacillus velezensis DSYZ, a Plant Growth-Promoting Rhizobacterium with Antifungal Properties

**DOI:** 10.1128/MRA.01217-18

**Published:** 2019-02-21

**Authors:** Jian Zhao, Hu Liu, Kai Liu, Hao Li, Yulong Peng, Jing Liu, Xiaobin Han, Xin Liu, Liangtong Yao, Qihui Hou, Chengqiang Wang, Yanqin Ding, Binghai Du

**Affiliations:** a College of Life Sciences and Shandong Key Laboratory of Agricultural Microbiology and National Engineering Laboratory for Efﬁcient Utilization of Soil and Fertilizer Resources, Shandong Agricultural University, Taian, China; b Zunyi Tobacco Monopoly Administration of Guizhou, Zunyi, China; c Middle School Attached to Beijing Medical University, Beijing, China; University of Maryland School of Medicine

## Abstract

Bacillus velezensis DSYZ is a plant growth-promoting rhizobacterium with the capacity to control fungal pathogens. It was isolated from the rhizosphere soil of garlic. Here, we present the complete genome sequence of B. velezensis DSYZ. Several gene clusters that are related to the biosynthesis of antimicrobial compounds were predicted.

## Abstract (precis)

Bacillus velezensis DSYZ is a plant growth-promoting rhizobacterium with the capacity to control fungal pathogens. It was isolated from the rhizosphere soil of garlic.

## ANNOUNCEMENT

Plant growth-promoting rhizobacteria (PGPR) are a group of rhizosphere bacteria which can improve plant growth by inducing plant disease resistance, phytohormone production, siderophore production, phosphate solubilization, and biological nitrogen fixation (1–4). As a member of PGPR, Bacillus velezensis possesses abilities to improve plant growth and could be used in agriculture, for example, to control the growth of many pathogenic microorganisms by producing secondary metabolites or antibiotics (5–8). B. velezensis ZSY-1 was reported to suppress the growth of Alternaria solani and Botrytis cinereal (9). B. velezensis Y6 and F7 have potential as biocontrol agents against Ralstonia solanacearum and Fusarium oxysporum (10). B. velezensis KLP2016 showed growth inhibition against Aspergillus niger and *Mucor* spp. (11). B. velezensis JTYP2 exhibited strong inhibition activity against F. inflexum, which can cause Echeveria laui black rot (8). In addition, B. velezensis showed the ability to promote plant growth (12).

B. velezensis DSYZ was isolated from garlic rhizosphere soil in Jining, China. The soil was diluted and then spread on Luria-Bertani (LB) medium to select single colonies at 37°C (13, 14). Then colonies were tested for the ability to antagonize the growth of pathogenic microorganisms with plate confrontation assays. Strain DSYZ was recognized as an effective PGPR by suppressing the growth of F. oxysporum (15) and F. moniliforme (16), which can cause soilborne diseases in many plants.

The genomic DNA was extracted with phenol and chloroform and then ethanol precipitated. The complete genome of DSYZ was sequenced using the Illumina MiSeq and PacBio platforms. The Illumina genome sequencing library was constructed according to the standard Illumina TruSeq Nano DNA LT sample preparation guide (17). The PacBio genome sequencing library was constructed according to a 20 K library strategy (18). A 20-kb insert library was prepared and sequenced on one SMRT cell (19). Using the Illumina platform, the adapters at the 3′ end were removed with AdapterRemoval (v2.1.7). Based on k-mer frequency, SOAPec (v2.0) software was used to calibrate all reads. The k-mer used in calibration was set to 17. On the PacBio platform, according to the coverage, seed reads and short reads were distinguished. Using the Arrow algorithm, we corrected seed reads with short reads and then stitched the resulting seed reads (20). A total of 6,331,672 original reads were obtained with the Illumina platform. Up to 6,297,064 high-quality reads totaling 1,812,964,106 bases were obtained by filtration. A total of 58,713 sequences were obtained with PacBio sequencing, with a length from 200 to 51,016 bp. Using the PacBio platform, the *N*_50_ value of raw sequences is 11,748 bp, and one contig was acquired. The sequencing coverage reached 425×. All reads were *de novo* assembled with HGAP4 (21) and Canu v1.6 (22). The genome sequence was annotated with the NCBI Prokaryotic Genome Annotation Pipeline (PGAP) (https://www.ncbi.nlm.nih.gov/genome/annotation_prok/). We simultaneously analyzed the secondary metabolism clusters in the complete genome with antiSMASH (v3.0.5l, http://antismash.secondarymetabolites.org/) (23).

B. velezensis DSYZ contains a 4,258,978-bp chromosome and a 62,485-bp plasmid, with a G+C content of 45.78% and 40.31%, respectively. A total of 4,363 genes were annotated with PGAP, including 4,143 coding genes, 27 rRNAs, 86 tRNAs, 5 ncRNAs, and 102 pseudogenes. The analysis of secondary metabolites showed that 13 gene clusters relevant to producing active antagonistic or other useful substances were predicted. Among them, five gene clusters showed 100% similarity with recognized biosynthetic gene clusters, and they were identified as producing fengycin, bacillibactin, bacillaene, macrolactin, and bacilysin. The other gene clusters were predicted to produce, for example, rhizocticin, butirosin, surfactin, terpenes, and difficidin. The complete genome sequence of B. velezensis DSYZ will contribute to revealing the molecular mechanisms of this species for promotion of plant growth and should provide benefits in applications for biological control in agriculture.

### Data availability.

The complete genome sequence of B. velezensis DSYZ has been deposited in GenBank under the accession numbers CP030150 (chromosome) and CP030151 (plasmid). The raw sequences from the Illumina platform have been deposited in the SRA under the accession number SRR8252721.
